# Physical Competence, Physical Well-Being, and Perceived Physical Literacy among Older Adults in Day Care Centers of Hong Kong

**DOI:** 10.3390/ijerph19073851

**Published:** 2022-03-24

**Authors:** Yan Huang, Raymond K. W. Sum, Yi-Jian Yang, Nelson C. Y. Yeung

**Affiliations:** 1 Department of Sports Science and Physical Education, The Chinese University of Hong Kong, Hong Kong 999077, China; hayleyhy@link.cuhk.edu.hk (Y.H.); yyang@cuhk.edu.hk (Y.-J.Y.); 2 The Jockey Club School of Public Health and Primary Care, The Chinese University of Hong Kong, Hong Kong 999077, China; nelsonyeung@cuhk.edu.hk

**Keywords:** physical competence, physical well-being, perceived physical literacy, older adults, day care center, The COVID-19 pandemic

## Abstract

In Hong Kong, where the aging problem is inevitable, it is increasingly common for older adults to be admitted to day care centers. However, there has been limited research exploring conceivable indicators of healthy aging among older adults in such settings. The present study investigated the associations among the three indicators (physical competence, physical well-being, and perceived physical literacy) among older adults in day care centers of Hong Kong. A total of 97 participants (aged 60 years old or above) participated in the study from April to July 2021 amid the COVID-19 pandemic. Data on participants’ sociodemographic information, physical competence (PC), physical well-being (PWB), and perceived physical literacy (PPL) were collected. Our results showed that the level of PC reached a high level among the participants. Positive correlations were found between PC and PWB and between PPL and PWB (r = 0.22–0.23, *p* < 0.05). However, PC was not associated with PPL (r = 0.11, *p* > 0.05). In addition, as a component within PPL, “knowledge and understanding” (KU) was found to be correlated with PC (r = 0.21, *p* < 0.05) and had a positive and moderate correlation with PWB (r = 0.35, *p* < 0.01). The results suggest that older adults admitted to day care centers maintain and enhance their physical competence to improve their physical well-being. Greater knowledge and understanding of physical literacy and physical health should be delivered among day care centers considering future development.

## 1. Introduction

Population aging has become a global health issue due to the combination of decreasing birth rates and increasing life expectancies. According to World Population Prospects 2019, there will be one in six people over the age of 65, worldwide, by 2050. Hong Kong ranks among the top five countries or regions in the world with the largest increase in the proportion of elderly people aged 65 or over between 2019 and 2050 [1]. In Hong Kong, the percentage of people aged 60 years or over has been projected to account for 33.6% of the total population by 2030, and according to the Hong Kong Population Projection, this proportion is estimated to increase to 39.3% by 2060 [2]. Public sections and pertinent stakeholders should contribute accordingly, and other services for reinforcing older adults’ healthy aging (e.g., nursing homes, multiservice centers, day care centers) should also be addressed [3].

Recently, day care centers for older adults have been focused on and discussed in relation to issues in public health [4]. Currently, there is a global consensus that day care centers for the elderly can help reduce the need for clinics or residential care for older people [5]. In 2012, the number of adult day care centers throughout the US was reported to have reached over 4600 and appeared to be growing consistently [6]. With more day care centers for the elderly being established, more exploration of the experiences among this population needs to be conducted [7]. The continuing investigation on issues regarding day care centers for older adults is also apparent in the UK, where considerations have been raised regarding the different needs of aging older adults [8].

It has become increasingly common for older adults to register at day care centers in Hong Kong. As of 1 October 2021, there were 91 registered and 71 self-financed day care centers for the elderly across Hong Kong, based on the report from the Social Welfare Department [9]. Older adults, aged 60 or above, living in the community and not receiving institutional service, could be recommended for these community care services under the Standardized Care Need Assessment Mechanism for Elderly Services, which is comprehensive, including applicants’ abilities in coping with daily activities, behavior and emotion, communication skills, etc. In addition, elderly individuals could be referred to these center services by other social agencies, district groups, and social workers. These center-based community care services provide daytime care and rehabilitation exercises. They also promote social activities for the elderly to enable them to develop their personal interests and potential, through different activities and groups, and achieve active aging. Nonetheless, there is still limited research and understanding regarding the physical characteristics and features of older adults from this setting in Hong Kong.

The World Health Organization [10] has proclaimed 2021–2030 to be the Decade of Healthy Aging, with an emphasis that the participation of older adults is central to, and fully engaged in, the initiatives to promote healthy aging. These initiatives strongly encourage the physically literate elderly, who can maintain physical competence and physical well-being, to perform activities they value and make changes to their environment. 

Afforded by an individual’s ability, physical competence (PC) was defined as proficiency in movements and capacities [11]. An updated definition further includes the movement patterns that interact within a wide range of environments [12]. PC is a fundamental aspect of being human and also lies at the center of the value of “Education and public health agenda—physical literacy [13]”. 

Physical literacy can be defined as the motivation, confidence, physical competence, knowledge, and understanding to value and take responsibility for engaging in physical activities for life [14]. Furthermore, the ongoing commitment and lifelong engagement in physical activities are highly connected and emphasize individualization, according to one’s endowment. Hence, physical literacy is an inclusive concept, containing physical, affective, and cognitive domains. In practice, it is difficult to have a unified and comprehensive standard to measure physical literacy. Previous research showed the possibility of exploring one’s perceived physical literacy (PPL) [15]. PPL is a self-reflective perception and is considered to be a significant indicator to be explored throughout the course of an individual’s life. 

There is no consensus to the definition of well-being, though it broadly refers to overall positive emotions and moods. Physical well-being (PWB) is regarded as a critical indicator of overall well-being [16]. PWB refers to the capability of performing physical activities and the ability to conduct social roles without being hindered by physical limitations, pain, or biological health indicators [17]. PWB could refer to feeling very healthy and full of energy, etc. Since the definition of PWB has more than one layer, a range of factors might be expected when associating it with older adults. Research has shown that physical activities influence one’s well-being and have positive effects on PWB [18]. However, there is a lack of research exploring the relationships among PWB, PC, and PPL among the elderly, especially in the day care center setting.

Amid the COVID-19 pandemic, public health agencies worldwide have come to a consensus that older adults are the demographic with the highest rates of mortality [19]. Individuals who are old and have complex health conditions are highly susceptible to the virus [20]. A further investigation stated that physical inactivity was associated with a higher risk for severe virus outcomes among older adults [21]. During the pandemic, social distancing, quarantines, and lockdowns posed challenges for remaining physically active [22]. In addition, physical inactivity is strongly linked to mobility limitations, which further increase older adults’ risk of falls [23]. To older adults, falls and fall-related hip fractures are major concerns [24], have negative impacts on the older adults’ quality of life, and tend to overwhelm health care systems [25]. This burden on the health care system may increase continuously if the pandemic persists for a long time. Moreover, research showed a profoundly negative impact of physical inactivity on the psychological health and well-being of people in Italy [26]. The situation is similar in Hong Kong, where day care centers have been affected by the pandemic. Social activities are decreased, and the maximum number of people permitted in social gatherings has been constantly adjusted according to the government’s epidemic prevention policy. One recent study reported anxiety symptoms among a moderate proportion of nurses in Hong Kong amid the COVID-19 pandemic [27]. Consequently, it is important for researchers to further explore conceivable indicators of healthy aging among older adults, especially in the continuing aging and pandemic-affected society of Hong Kong.

After synthesizing the background and literature reviews, this study aimed to explore the correlation between PC, PWB, and PPL among older adults in day care centers of Hong Kong. The hope is to fill the research gap and provide further understanding regarding the physical characteristics and features of the elderly in day care centers of Hong Kong. We hypothesized that PC would be positively associated with both PWB and PPL and that PWB would be positively associated with PPL.

## 2. Methods

### 2.1. Study Design

This was a cross-sectional study conducted from April to July 2021 in Hong Kong.

Participants were recruited from day care centers across Hong Kong. Inclusion criteria were: (1) aged above 60 years and registered in one day care center for the elderly in Hong Kong; (2) able to walk eight meters without assistance; (3) able to speak and understand Cantonese and/or Mandarin; (4) able to provide informed consent on their own behalf. Recruitment was conducted through posters and leaflets, followed by setting up counters in the participating day care centers, which was supported by the social workers of the centers. Those who expressed interest and met the inclusion criteria were invited to participate. Written informed consent was obtained. Approval for the use of human subjects was obtained from the University Survey and Behavioral Research Ethics Committee of the Faculty of Education of the Chinese University of Hong Kong (ref. no. SBRE-20-238).

This study was conducted at one of the sensory integration training centers in Hong Kong. Students and social workers volunteered to serve as helpers for distributing and collecting questionnaires, while the objective measurements were led by certified physical instructors from The Physical Fitness Association of Hong Kong, China.

Since the study was conducted during the COVID-19 pandemic, participants were divided into small groups to abide by the regulations of social distancing. All participants and people involved in the measurements were reminded to wear a mask during the fieldwork. All equipment used in the measurements was sterilized before its next use.

### 2.2. Measures

#### 2.2.1. Sociodemographic Information

Sociodemographic information was collected from all the participants by self-report, including age, body height (cm), body weight (kg), body mass index (BMI) (kg/m^2^), gender, education attainment (no formal education, primary school education, high school education, university education or above), and living status (living alone or living with others).

#### 2.2.2. Physical Competence

The Short Physical Performance Battery (SPPB) [28] was adopted to measure participants’ physical competence. This kit of measurements assessed complex capabilities (strength, mobility, balance, flexibility, and aerobic fitness) of older adults and included three objective measurements: (1) gait speed test, (2) chair stand test, and (3) three individual balance tests. The balance tests included were a side-by-side stand, a semi-tandem stand, and a tandem stand. The SPPB summary scores ranged from 0 to 12, with higher scores indicating better performance. According to a recent scoping review of measurements of older adults’ PC under the concept of physical literacy [29], SPPB was reported to have excellent test–retest reliability, especially for community-dwelling older adults.

#### 2.2.3. Physical Well-Being

PWB was measured by a subscale of Perceived Well-being Scale [30]. The 14-item scale is an index of general well-being and includes two subscales: psychological well-being (six items) and physical well-being (eight items). Example items included (a) “I am in good shape physically” and (b) “I don’t have many physical complaints”. Participants rated each item on a 7-point Likert scale (from 1—strongly disagree to 7—strongly agree). A higher score on each item reflected a higher level of well-being. Within the limited measures available to assess PWB among older adults, this scale was reported to be a short and convenient measure for the elderly, with ecological validity regardless of the environment or setting [30].

#### 2.2.4. Perceived Physical Literacy

A Perceived Physical Literacy Instrument (PPLI) [15] was adopted to measure the PPL of the participants. This 9-item instrument was designed from previous evidence from research, and includes three subscales confirmed from focus group interviews: sense of self and self-confidence (SSSC), self-expression and communication with others (SECO), and knowledge and understanding (KU). Sample items included (a) “I am physically fit, in accordance with my age (SSSC)”, (b) “I have strong social skills (SECO)”, and (c) “I am aware of the benefits of sports related to health (KU)”. Participants responded on a 5-point Likert scale (from 1—strongly disagree to 5—strongly agree). A higher score on each item indicated a higher level of the PPL. The scales support factorial validity and were reliable, with an internal consistency ranging from 73 to 76 [31].

### 2.3. Data Analysis

SPSS version 26 for Windows was used for data analysis. Descriptive statistics were calculated to describe participants’ sociodemographic information and the study variables. Pearson’s product–moment correlation coefficients (r) were calculated to examine the correlations between physical competence, physical well-being, and perceived physical literacy. Two-tailed *p* < 0.05 was set to define the statistical significance in this study.

## 3. Results

A total of 97 participants were recruited for this study. Their demographic and sociodemographic information are listed in Table 1 and Table 2. Their mean age was 69.4 years (SD = 3.7 years), with 35.1% within the age range of 65–69 years. The mean (SD) BMI among the participants was reported as 23.7 (3.4).

The majority of the participants were women (75.3%). For education attainment, nearly a third (62.9%) of participants had a high school education or greater, while 4.1% had received no formal education at all. For living status, 23.7% of the participants reported that they lived alone, while 76.3% reported living with someone else.

The mean (SD) values of the outcome variables are listed in Table 3. The participants’ reported PPL was an average of 35.4 (4.7). For the subscales, the mean (SD) values of SSSC and SSCO were 11.4 (1.9) and 10.9 (2.2), respectively, which were lower than the component of KU, which had a mean value of 13.1 (1.4). The mean (SD) of PWB was reported to be 35.3 (4.7). The PC scores had a mean (SD) of 11.5 (0.8), which indicated a high level of performance.

There was a weak positive correlation between PWB and PC, r (97) = 0.22, *p* < 0.05 (Table 3). A weak correlation was found between PWB and PPL, r (97) = 0.23, *p* < 0.05. There was a moderate positive correlation between PWB and KU within PPL, r (97) = 0.35, *p* < 0.01. In regard to the relationship between PPL and PC, there was no significant correlation (r = 0.11, *p* > 0.05). However, KU within PPL was significantly associated with PC, r (97) = 0.21, *p* < 0.05 (Table 3).

## 4. Discussion

The present study measured PC, PWB, and PPL among day care centers’ older adults in Hong Kong, revealing correlations between them. Except for the relationship between PC and PPL, the results were consistent with what we hypothesized.

Our findings showed that PC of day care centers’ older adults in Hong Kong reached a high level, which indicated a lower risk of falls and death [32]. The services are community-based in Hong Kong, and findings were in line with previous empirical studies [33,34] that demonstrated the importance of maintaining high physical performance in preventing falls and deaths among the community-dwelling or hospitalized elderly. According to the results, the mean (SD) value of BMI among the participants was 23.7 (3.4), which was considered normal for older adults [35]. Based on one’s height and weight, individuals with high BMI levels are at greater risk of diseases such as cardiovascular disease [36], while a normal BMI level is associated with a good fitness level. This may explain the high performance score of PC in our study. 

In 2017, the life expectancies in Hong Kong increased to 81.9 years for men and 87.6 years for women [37], and have since reached 82.9 and 88.0 years, respectively [2]. Comprehensive tobacco control measures from legislation have contributed greatly to health promotion in Hong Kong. The low prevalence of smoking has been identified as the strongest determinant of the population’s longevity [38]. According to the previous systematic review, non-smokers tended to be more physically active than adults who smoke [39]. To some extent, the high population density of Hong Kong (6620 persons per km^2^ in 2012) meant that space needed to be optimized, including the allocation of green exposure within the urban areas. This green exposure impacts the population’s motivation and is associated with greater engagement in walking or other physical activities in Hong Kong [40]. Studies have demonstrated that street greenery, especially the parks in the city, contributes to the walking time and physical activity of older adults in Hong Kong [41,42]. Participants in our study tended to be at an earlier stage in their journeys to aging compared to older adults from other countries and areas (who were eligible for the assessment settings of our study). Active engagement in physical activity indicated better functional physical performances in our participants [43], which could be a plausible explanation for the high score of PC attained in this study. Another possibility is the sample selection bias: as these participants voluntarily joined this study, they may have had a higher level of fitness and were more motivated.

This study demonstrated that PC was positively associated with PWB. Previous research examined the relationship between PC and psychological well-being, and those findings explained that movement capability and engagement in physical activity were associated with the psychological well-being of older adults [44,45]. In Hong Kong, a recent study showed that physical competence was positively associated with subjective well-being [46]. While our study was the first to explore the correlation between PC and PWB, future studies are encouraged to enhance PWB through facilitating PC in future implementations. PWB was further found to be correlated with PPL. This correlation is of significant note for policymakers or pertinent stakeholders who may foster PPL in older adults and design programs for the development of physical literacy.

PC was not found to be associated with PPL. Physical literacy consisted of a blend of attributes, which indicated that the measurement of this indicator should adopt a comprehensive tool, consistent with a previous scoping review regarding the issue [29]. Meanwhile, in Hong Kong, physical literacy has been emphasized and practiced in the education system. However, there are a limited number of studies focused on older adults’ physical literacy to date. The concept and exploration of physical literacy among older adults in Hong Kong need to be enhanced [47].

To further discuss the components of PPL, our results also showed that the scores of SSSC and SSCO were lower than KU. The aspects with lower scores indicated that perceptions, attitudes, and the barriers behind them merit further exploration. Interestingly, KU was found to be a significant indicator, which was associated with PC and had a positive and moderate correlation with PWB. There was a large proportion of participants with a high school education attainment or greater, which complies with the previous finding that higher education is associated with better quality of life and well-being [48,49]. In addition, the aforementioned implementation and development of promoting physical literacy in Hong Kong should continue to be encouraged.

## 5. Conclusions

The current study is subject to limitations. Due to the COVID-19 pandemic, the motivation and willingness to engage in public activities may have decreased, and a larger sample size in the future may enhance the findings. The voluntary nature of the participation in the study may result in sample selection bias. The participants included more women than men, which may influence the results as well. Other issues may include the self-report data in the sociodemographic information.

However, the present study was the first to examine the relationships between PC, PWB, and PPL among older adults in day care centers of Hong Kong, where the population of elderly individuals is growing rapidly. Further exploration is still needed to address, stress, and solve issues relating to this population.

In conclusion, this study explored the relationship between PC, PWB, and PPL among older adults in day care centers of Hong Kong. Our findings showed a high level of PC for the participants. Positive correlations were found between PC and PWB, as well as PPL and PWB. There was no significant association between PC and PPL. However, as a component within PPL, KU was found to be correlated with PC and to have a positive and moderate correlation with PWB. This study emphasized the significant indicators for healthy aging and provided a further understanding of the physical characteristics and features of the elderly in day care centers of Hong Kong. Based on the results, we suggest that older adults, especially those admitted to day care centers, should maintain and enhance their physical competence to improve their physical well-being. Recommendations should include targeted and specific exercises, which consist of adequate balance, strength, and mobility training [50]. It is critical for them to make physical adaptations in the active aging process, especially when facing challenges that come with chronic disease, as well as maintaining their physical ability to interact with the environment. Greater knowledge and understanding of physical literacy and physical health should be delivered among day care centers considering the future development of health promotion.

## Figures and Tables

**Table 1:** Demographic characteristics of participants.

Variables	Mean ± SD	By Gender	
		Man	Woman
Age	69.4 ± 3.4	70.2 ± 4.9	69.2 ± 5.5
Body height (cm)	154.9 ± 8.1	164.2 ± 6.2	151.8 ± 6.2
Body weight (kg)	57.2 ± 10.8	66.4 ± 9.3	54.2 ± 9.5
Body mass index (BMI) (kg/m^2^)	23.7 ± 3.4	24.6 ± 2.9	23.5 ± 3.6

**Table 2:** Sociodemographic characteristics of participants.

Variables	n (%)	By Gender	
		Man	Woman
Age group			
60–64	21 (21.6)	3 (12.5)	18 (24.7)
65–69	34 (35.1)	8 (33.3)	28 (35.6)
70–74	27 (27.8)	10 (41.7)	17(23.3)
75–80	15 (15.5)	3 (12.5)	12 (16.4)
Education attainment			
No formal education	4 (4.1)	0 (0)	4 (5.5)
Primary school education	16 (16.5)	2 (8.3)	14 (19.2)
High school education	61 (62.9)	16 (66.7)	45 (61.6)
University education or above	16 (16.5)	6 (25.0)	10 (13.7)
Living status			
Living alone	23 (23.7)	3 (12.5)	20 (27.4)
Living with someone	74 (76.3)	21 (87.5)	53 (72.6)

**Table 3:** Pearson correlation coefficients among the studied independent and dependent variables.

Variables	Mean (SD)	1	2	3	4	5	6
1. Perceived physical literacy (PPL)	35.4 ± 4.7	1					
2. Sense of self and self-confidence (SSSC)	11.4 ± 1.9	0.895 **	1				
3. Self-expression and communication with others (SECO)	10.9 ± 2.2	0.866 **	0.688 **	1			
4. Knowledge and understanding (KU)	13.1 ± 1.4	0.753 **	0.572 **	0.472 **	1		
5. Physical well-being (PWB)	35.3 ± 4.1	0.232 *	0.169	0.123	0.354 **	1	
6. Physical competence (PC)	11.5 ± 0.8	0.114	0.098	0.024	0.207 *	0.222 *	1

* *p* < 0.05; ** *p* < 0.01.

## Data Availability

The data presented in this study are available on request from the corresponding author. The data are not publicly available due to privacy or ethical restritions.

## References

[1] United Nations Department of Economic and Social Affairs Population Division (2019). World Population Ageing 2019. World Population Ageing 2019: Vol. Highlights.

[2] Census & Statistics Department Life Expectancy at Birth (Male and Female), 1971–2020. https://www.chp.gov.hk/en/statistics/data/10/27/111.html.

[3] Cheung J.C.K., Kwan A.Y.H., Chan S.S.C., Ngan R.M.H., Ng S.H., Leung E.M.F., Lau A. (2005). Quality of Life in Older Adults: Benefits from Caring Services in Hong Kong. Soc. Indic. Res..

[4] Piercy K.W., Blieszner R. (1999). Balancing Family Life: How Adult Children Link Elder-Care Responsibility to Service Utilization. J. Appl. Gerontol..

[5] Fields N.L., Anderson K.A., Dabelko-Schoeny H. (2014). The effectiveness of adult day services for older adults: A review of the literature from 2000 to 2011. J. Appl. Gerontol. Off. J. South. Gerontol. Soc..

[6] Anderson K.A., Dabelko-Schoeny H., Johnson T.D. (2012). The State of Adult Day Services: Findings and Implications From the MetLife National Study of Adult Day Services. J. Appl. Gerontol..

[7] Dubus N. (2015). A Qualitative Study of Older Adults and Staff at an Adult Day Center in a Cambodian Community in the United States. J. Appl. Gerontol..

[8] Manthorpe J., Moriarty J. (2014). Examining day centre provision for older people in the UK using the Equality Act 2010: Findings of a scoping review. Health Soc. Care Community.

[9] Social Welfare Department Day Care Centres/Units for the Elderly. https://www.swd.gov.hk/en/index/site_pubsvc/page_elderly/sub_csselderly/id_daycarecen/.

[10] WHO/Malin Bring Decade of Healthy Ageing 2021–2030. https://www.euro.who.int/en/health-topics/Life-stages/healthy-ageing/news/news/2021/01/decade-of-healthy-ageing-2021-2030.

[11] Whitehead M. (2010). Glossary. Physical Literacy: Throughout the Lifecourse.

[12] Whitehead M. (2019). Charting the physical literacy journey. Physical Literacy across the World.

[13] Almond L., Whitehead M. (2010). Physical literacy and the adult population. Physical Literacy: Throughout the Lifecourse.

[14] Whitehead M. (2019). Definition of physical literacy. Physical Literacy across the World.

[15] Sum R.K.W., Cheng C., Wallhead T., Kuo C., Wang F., Choi S. (2018). Perceived physical literacy instrument for adolescents: A further validation of PPLI. J. Exerc. Sci. Fit..

[16] Andrews F.M., Frank M., Withey S.B. (1976). Social Indicators of Well-Being: Americans’ Perceptions of Life Quality.

[17] Capio C.M., Sit C.H.P., Abernethy B., Michalos A.C. (2014). Physical Well-Being BT-Encyclopedia of Quality of Life and Well-Being Research.

[18] Penedo F.J., Dahn J.R. (2005). Exercise and Well-Being: A Review of Mental and Physical Health Benefits Associated with Physical Activity. Curr. Opin. Psychiatry.

[19] Comas-Herrera A. (2020). Mortality Associated with COVID-19 Outbreaks in Care Homes: Early International Evidence. https://ltccovid.org/wp-content/uploads/2020/04/Mortality-associated-with-COVID-12-April-4.pdf.

[20] Chu C.H., Donato-Woodger S., Dainton C.J. (2020). Competing crises: COVID-19 countermeasures and social isolation among older adults in long-term care. J. Adv. Nurs..

[21] Sallis R., Young D.R., Tartof S.Y., Sallis J.F., Sall J., Li Q., Smith G.N., Cohen D.A. (2021). Physical Inactivity is Associated with a Higher Risk for Severe COVID-19 Outcomes: A Study in 48 440 Adult Patients. Br. J. Sports Med..

[22] Woods J.A., Hutchinson N.T., Powers S.K., Roberts W.O., Gomez-Cabrera M.C., Radak Z., Berkes I., Boros A., Boldogh I., Leeuwenburgh C. (2020). The COVID-19 pandemic and physical activity. Sports Med. Health Sci..

[23] Rosenberg D.E., Bombardier C.H., Hoffman J.M., Belza B. (2011). Physical Activity among Persons Aging with Mobility Disabilities: Shaping a Research Agenda. J. Aging Res..

[24] Yang Y., Komisar V., Shishov N., Lo B., Korall A.M.B., Feldman F., Robinovitch S.N. (2020). The Effect of Fall Biomechanics on Risk for Hip Fracture in Older Adults: A Cohort Study of Video-Captured Falls in Long-Term Care. J. Bone Miner. Res..

[25] Scott V., Wagar L., Elliott S. (2010). Falls and related injuries among older Canadians: Fall-related hospitalizations and intervention initiatives. Prepared on Behalf of the Public Health Agency of Canada, Division of Aging and Seniors.

[26] Maugeri G., Castrogiovanni P., Battaglia G., Pippi R., D’Agata V., Palma A., Di Rosa M., Musumeci G. (2020). The impact of physical activity on psychological health during COVID-19 pandemic in Italy. Heliyon.

[27] Yeung N.C., Wong E.L., Cheung A.W., Yeoh E., Wong S.Y. (2021). Feeling Anxious Amid the COVID-19 Pandemic: Factors Associated with Anxiety Symptoms Among Nurses in Hong Kong. Front. Psychol..

[28] Guralnik J.M., Simonsick E.M., Ferrucci L., Glynn R.J., Berkman L.F., Blazer D.G., Scherr P.A., Wallace R.B. (1994). A short physical performance battery assessing lower extremity function: Association with self-reported disability and prediction of mortality and nursing home admission. J. Gerontol..

[29] Huang Y., Sum K.W.R., Yang Y.J., Yeung N.C.Y. (2020). Measurements of older adults’ physical competence under the concept of physical literacy: A scoping review. Int. J. Environ. Res. Public Health.

[30] Reker G.T., Wong P.T.P. (1984). Psychological and Physical Well-Being in the Elderly: The Perceived Well-Being Scale (PWB). Can. J. Aging/La Rev. Can. Du Vieil..

[31] Choi S.M., Sum R.K.W., Leung E.F.L., Ng R.S.K. (2018). Relationship between perceived physical literacy and physical activity levels among Hong Kong adolescents. PLoS ONE.

[32] Przkora R., Kinsky M.P., Fisher S.R., Babl C., Heyde C.E., Vasilopoulos T., Kaye A.D., Volpi E. (2019). Functional Improvements Utilizing the Short Physical Performance Battery (SPPB) in the Elderly after Epidural Steroid Injections. Curr. Pain Headache Rep..

[33] Arnau A., Espaulella J., Méndez T., Serrarols M., Canudas J., Formiga F., Ferrer M. (2016). Lower limb function and 10-year survival in population aged 75 years and older. Fam. Pract..

[34] Fisher S., Ottenbacher K.J., Goodwin J.S., Graham J.E., Ostir G.V. (2009). Short Physical Performance Battery in hospitalized older adults. Aging Clin. Exp. Res..

[35] Swanton K. (2008). Healthy Weight, Healthy Lives: A toolkit for developing local strategies. Lond. Dep. Health.

[36] Bray G.A. (1992). Pathophysiology of obesity. Am. J. Clin. Nutr..

[37] Chung R.Y.-N., Marmot S.M. (2020). People in Hong Kong Have the Longest Life Expectancy in the World: Some Possible Explanations. NAM Perspect.

[38] Ni M.Y., Canudas-Romo V., Shi J., Flores F.P., Chow M.S.C., Yao X.I., Ho S.Y., Lam T.H., Schooling C.M., Lopez A.D. (2021). Understanding longevity in Hong Kong: A comparative study with long-living, high-income countries. Lancet Public Health.

[39] Kaczynski A.T., Manske S.R., Mannell R.C., Grewal K. (2008). Smoking and physical activity: A systematic review. Am. J. Health Behav..

[40] Lu Y., Sarkar C., Xiao Y. (2018). The effect of street-level greenery on walking behavior: Evidence from Hong Kong. Soc. Sci. Med..

[41] Wagner P., Duan Y.P., Zhang R., Wulff H., Brehm W. (2020). Association of psychosocial and perceived environmental factors with park-based physical activity among elderly in two cities in China and Germany. BMC Public Health.

[42] Yang L., Liu J., Liang Y., Lu Y., Yang H. (2021). Spatially Varying Effects of Street Greenery on Walking Time of Older Adults. ISPRS Int. J. Geo-Inf..

[43] Ma D.Y., Wong C.H.Y., Leung G.T.Y., Fung A.W.T., Chan W.C., Lam L.C.W. (2017). Physical Exercise Helped to Maintain and Restore Functioning in Chinese Older Adults With Mild Cognitive Impairment: A 5-Year Prospective Study of the Hong Kong Memory and Ageing Prospective Study (HK-MAPS). J. Am. Med. Dir. Assoc..

[44] Osada H., Shabata H., Watanabe S., Kumagai S., Suzuki T. (2000). The Relationship between Psychological Well-Being and Physical Functioning in Japanese Urban and Rural Older Adults. J. Aging Phys. Act..

[45] Veldema J., Jansen P. (2019). The Relationship among Cognition, Psychological Well-being, Physical Activity and Demographic Data in People over 80 Years of Age. Exp. Aging Res..

[46] Yu Y., Fong V.W.I., Lau J.T.F., Sum R.K.W., Leung E.F.L., Mo P.K.H. (2020). The associations between psychological needs, health-related quality of life and subjective well-being among Chinese older people: A cross-sectional study. Health Soc. Care Community.

[47] Sum K.-W.R., Li M.-H., Choi S.-M., Huang Y., Ma R.-S. (2020). In/Visible Physical Education and the Public Health Agenda of Physical Literacy Development in Hong Kong. Int. J. Environ. Res. Public Health.

[48] Borders T.F., Aday L.A., Xu K.T. (2004). Factors associated with health-related quality of life among an older population in a largely rural western region. J. Rural. Health Off. J. Am. Rural. Health Assoc. Natl. Rural. Health Care Assoc..

[49] Zahran H.S., Kobau R., Moriarty D.G., Zack M.M., Holt J., Donehoo R. (2005). Health-related quality of life surveillance--United States, 1993-2002. Morb. Mortal. Wkly. Report. Surveill. Summ..

[50] Roetert E.P., Ortega C. (2019). Physical literacy for the older adult. Strength Cond. J..

